# Single tin-vacancy center in nanoscale diamond

**DOI:** 10.1186/s11671-025-04256-0

**Published:** 2025-05-14

**Authors:** Masanori Fujiwara, Masanao Ohori, Frederick Tze Kit So, Yuto Makino, Naoya Morioka, Izuru Ohki, Ryuji Igarashi, Masahiro Nishikawa, Norikazu Mizuochi

**Affiliations:** 1 Institute for Chemical Research, Kyoto University, Gokasho, Uji, Kyoto 611-0011 Japan; 2 Institute for Quantum Life Science, National Institutes for Quantum Science and Technology, Anagawa, Inage, Chiba 263-8555 Japan; 3 Research and Development Headquarters, Daicel Corporation, 1239, Shinzaike, Aboshi-Ku, Himeji, Hyogo 671-1283 Japan; 4 Center for Spintronics Research Network, Institute for Chemical Research, Kyoto University, Gokasho, Uji, Kyoto 611-0011 Japan; 5 School of Life Science and Technology, Institute of Science Tokyo, Ookayama, Meguro-ku, Tokyo 152-8550 Japan

**Keywords:** Tin-vacancy center, Detonation nanodiamond, Single-photon source

## Abstract

Group-IV color centers in diamonds are promising not only as spin–photon interfaces for quantum networks but also as multicolor photoluminescence probes and all-optical temperature sensors for biological research. Therefore, generating group-IV color centers in detonation nanodiamonds (DNDs), the smallest class of diamond nanocrystals, has become a research focal point. This approach holds potential for the noninvasive introduction of diamond sensors into living cell organelles, enabling sensing of local structural and temperature changes with minimal disturbance. Following the successful generation of silicon- and germanium-vacancy centers in DNDs, the generation of tin-vacancy (SnV) centers was investigated. Similar to other group-IV color centers, the SnV centers exhibit strong photoluminescence. In addition, the SnV center offers a long spin coherence time compared with those of silicon- and germanium-vacancy centers. However, the strain induced by large Sn atoms poses substantial challenges for generating SnV centers in ultrasmall nanodiamonds. In this study, Sn-doped DNDs with a mean particle size of ~ 5 nm were subjected to a 3 days boiling acid treatment, which led to sharp zero-phonon lines of SnV centers at ~ 620 nm in the photoluminescence spectra. Photon autocorrelation measurements further revealed the presence of single SnV centers in these DNDs.

## Introduction

Nanodiamonds (NDs) containing color centers are excellent photoluminescence (PL) probes for biological research because of their low cytotoxicity and versatile surface modification capabilities [1–4]. In addition, single NDs with single color centers are expected to serve as single-photon sources for integrated quantum technology and nanophotonics [5–8]. Among various color centers, the nitrogen-vacancy (NV) color center is the most extensively studied [9]. The negatively charged NV center (NV^−^) enables optically detected magnetic resonance (ODMR) measurements using visible light and microwaves, facilitating the development of quantum sensing techniques for measuring parameters such as magnetic field [10], electric field [11], temperature [12], pressure [13], and pH [14], with a high dynamic range [15] at room temperature. Group-IV color centers—specifically, silicon-, germanium-, tin-, and lead-vacancy (SiV, GeV, SnV, and PbV, respectively) centers are the next-most promising candidates after NV centers [16, 17]. The group-IV color centers consist of a lattice divacancy with a group-IV atom located at the center. A *D*_3d_ inversion-symmetry structure ensures an intense and sharp zero-phonon line (ZPL) for all negatively charged group-IV color centers, even at room temperature. However, various parameters such as the ZPL peak wavelength, quantum efficiency, radiative lifetime, and spin coherence time vary depending on the group-IV atom [16, 17]. Under ambient conditions, their ZPL peak wavelengths shift linearly to longer wavelength with increasing temperature [18–20]. Because of these properties, the group-IV color centers are expected to be excellent candidates for multicolor PL probes and all-optical temperature sensors. Beyond their applications, group-IV color centers have also attracted attention as solid-state quantum nodes for quantum networks. They exhibit excellent potential to realize indistinguishable single-photon sources with high photon efficiency and possess a long spin coherence time suitable for entanglement generation between nodes at cryogenic temperatures [16, 17]. A recent study demonstrated a two-node quantum network based on SiV centers in nanophotonic cavities integrated with a telecommunication fiber network [21].

To date, larger NDs with a particle size of ~ 100 nm have been widely used in biological research to ensure sufficient brightness from an ensemble of color centers. Meanwhile, NDs of ~ 10 nm are crucial for imaging and sensing within living cells or cell organelles. For instance, the inner diameter of a mammalian nuclear pore is ~ 40 nm [22]. To enable noninvasive introduction into the nucleus, ND sensors should be smaller than a few tens of nanometers. Among the several methods in synthesizing NDs [23], our group focused on the detonation synthesis method [24], which produces NDs with particle sizes of ~ 10 nm at an industrial scale and at low cost [25]. The globular and uniform shape of DNDs makes them suitable for use in practical applications. Although the synthesis of DNDs with NV centers has been widely reported [26], the synthesis of DNDs with group-IV color centers has remained a substantial challenge until recently [27]. In 2021 and 2022, our group reported the first syntheses of SiV- [28] and GeV-containing DNDs [29] thanks to dopant molecules with group IV atoms centered on tetraphenyl or triphenyl groups. These DNDs, with mean particle sizes of ~ 20 nm, have also been used for all-optical thermometry [30, 31]. In addition, our group synthesized Sn-doped DNDs with the aim of generating SnV centers [29]. Elemental analysis using X-ray fluorescence confirmed the presence of Sn atoms in the DNDs. The equilibrium concentration ratio of SnV-DNDs to SiV-DNDs was estimated to be 0.01–0.02, based on the thermodynamic calculations of concentration ratio of Sn- to Si-containing droplet carbons, precursors of SnV- and SiV-DNDs, during the detonation process. Since SiV centers were confirmed at many observation points, SnV centers also could be observed, even though at a lower concentration. However, ZPLs of SnV^−^ centers, experimental evidence for their presence in DNDs, were not detected [29].

Like other group-IV color centers, the SnV^−^ center exhibits a sharp ZPL and can be used for all-optical thermometry [19]. In addition, the electron-spin coherence time for the SnV^−^ center is substantially longer than those for SiV^−^ and GeV^−^ centers because of its large spin–orbit coupling constant [32, 33]. Whereas typical quantum memory experiments using SiV^−^ or GeV^−^ centers require a diluted refrigerator (< 1 K) to suppress phonon-mediated decoherence, SnV^−^ centers can maintain their coherence time at a relatively high temperature (> 1 K) [17] and quantum memory experiments would be conducted without the diluted refrigerator. Therefore, SnV^−^ centers are more useful than SiV^−^ and GeV^−^ centers for both biological and quantum applications. However, creating SnV centers is more difficult than creating SiV and GeV centers, even in bulk diamonds, because the relatively large size of the Sn atom induces substantial strain and hinders the proper formation of SnV centers [33–37]. The estimated equilibrium concentration ratio of SnV-DNDs to SiV-DNDs also supports this difficulty [29]. In the case of microdiamonds, an early study performed the generation of SnV centers during the diamond synthesis from a Sn-graphite system using a high-pressure high-temperature (HPHT) technique [38]. In the case of NDs, to our knowledge, only one study has reported the successful generation of SnV centers [39]. The SnV-containing NDs were synthesized via a chemical vapor deposition technique onto a Sn-dopant-coated substrate and exhibited ZPLs of SnV^−^ centers. However, the mean particle size was not explicitly reported and appeared to considerably exceed 100 nm, as judged from the corresponding scanning electron microscope image. Therefore, generating SnV centers in ultrasmall NDs is still challenging.

In the current study, we investigate the presence of SnV centers in Sn-doped DNDs by improving the post-detonation process. Recently, numerous NV^−^ centers with stable and strong ODMR signals in both Si-doped and undoped DNDs were found after 3 days boiling acid treatments [40]. This treatment effectively removes surface residual *sp*^2^ carbon and metal impurities, leading to high charge stability of the NV^−^ centers in DNDs. Thus, we applied the same treatment to Sn-doped DNDs. To enhance the detection efficiency, we used a custom-made confocal microscope equipped with a band-bass filter targeting the ZPL of SnV^−^. With these improvements, we observed the ZPLs of SnV^−^ centers in Sn-doped DNDs and confirmed the presence of single SnV^−^s using photon autocorrelation measurements.

## Materials and methods

**Fig. 1:**
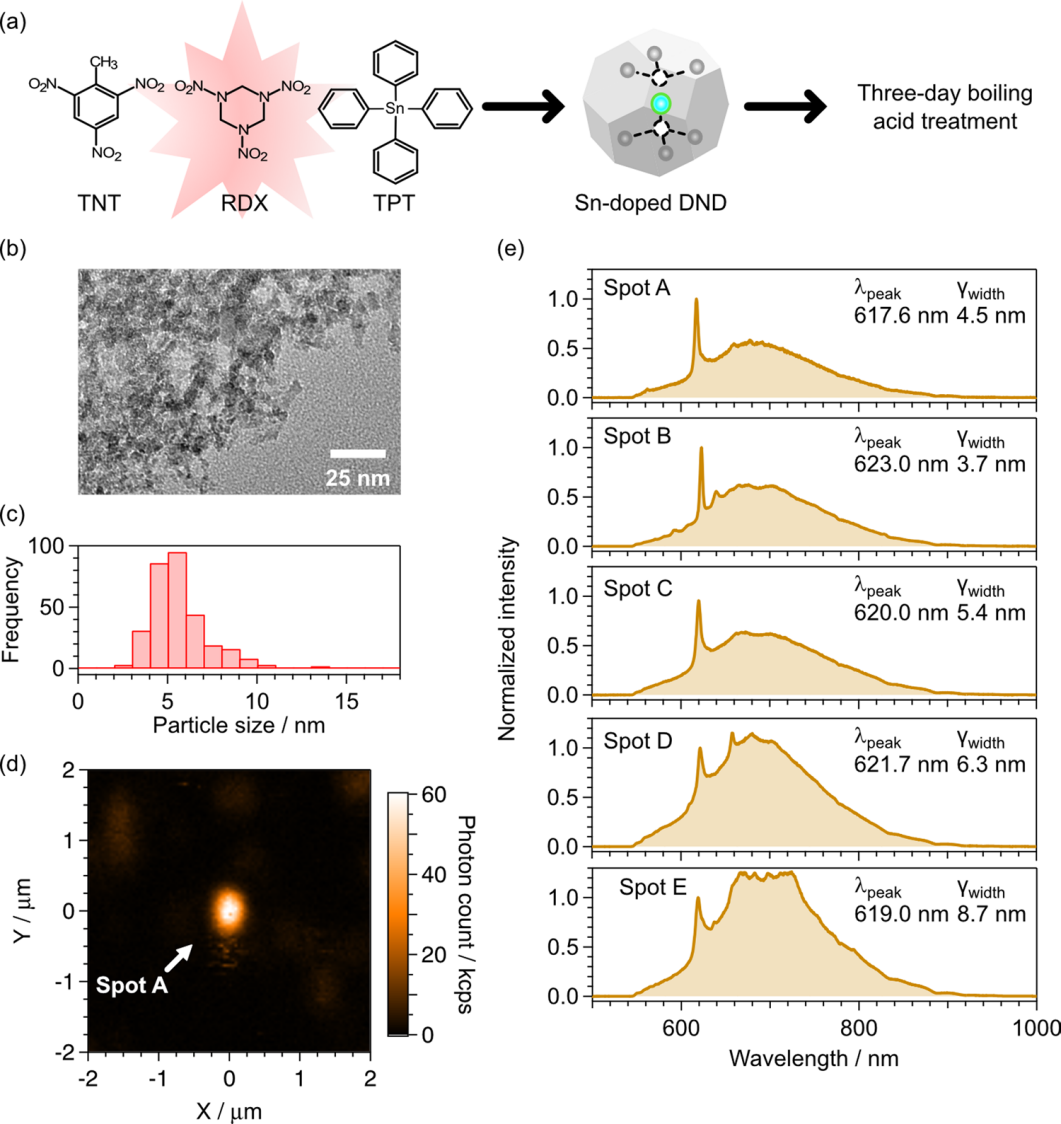
**a** Sample preparation of Sn-doped DNDs. **b** Typical TEM image. **c** Particle size distribution. **d** PL image measured with a 620 ± 5 nm band-pass filter. For the bright spot indicated by a white arrow (Spot A), the spot radius (*r*_*x*_, *r*_*y*_) becomes (270 nm, 370 nm), which is defined by a 1/e^2^ radius by a 2D Gaussian fitting. **e** Typical PL spectra with sharp peaks at a wavelength of 620 nm. The peak wavelength (*λ*_peak_) and the spectral width (*γ*_width_ = full-width at half-maximum) were obtained from a curve fitting using the sum of a Lorentzian, a linear term, and a constant term. The fitting wavelength range was fixed from 610 to 630 nm

The Sn-doped DND particles were prepared using the previously reported protocols [29]. A mixed explosive [2,4,6-trinitrotoluene (TNT) and hexahydro-1,3,5-trinitro-1,3,5-triazine (RDX)] with a Sn dopant [tetraphenyltin (TPT)] was detonated under a CO_2_ atmosphere [TNT / RDX / TPT = 59.2 / 39.6 / 1.2 (wt. %)] (Fig. 1a). The detonation products were purified with an acid mixture [HNO_3_ / H_2_SO_4_, / H_2_O = 9 / 76 / 15 (wt.%)] at 150 °C for 5 h. After cooling at 70 °C, the reaction mixtures were added to deionized (DI) water heated again at 150 °C for 5 h. The acid treatment was performed to remove *sp*^2^ carbons and metal impurities. The precipitates were then rinsed with DI water and dried. After drying, the products were treated with aqueous 8 M NaOH at 70 °C for 8 h to remove tin dioxides. Alkali-treated precipitates were added to DI water at room temperature, and the pH of the resultant mixtures was adjusted to 3–4 by addition of aqueous 1 M HCl. The suspension was centrifuged (CR22G, Hitachi Koki) at 8000 *g* for 10 min, and the precipitates were separated and added to DI water. The procedures of aqueous HCl addition and centrifugation were repeated. The collected precipitates were then rinsed with DI water and dried. The purified samples were air-oxidized [O_2_ / N_2_ = 4 / 96 (vol.%)] at 470 °C for 2 h to remove amounts of *sp*^2^ carbon.

After the air oxidization, the particle size was evaluated using a powder X-ray diffraction (XRD) pattern and transmission electron microscopy (TEM) images (Fig. 1b) [29]. The former revealed a mean particle size of 5.9 nm, calculated using Scherrer’s formula on the basis of the XRD peak corresponding to diamond’s (111) plane. The latter showed a particle size distribution centered at 4.7 nm with a standard deviation of 1.7 nm under the assumption of a normal distribution (Fig. 1c). Both results agree well with each other. Here, the contour of the DND particles in TEM images are determined manually. Overlapping particles are ignored, and only particles that appear to be single DND particles are counted. The size of a DND particle is determined by its maximum length, as defined by the contour [29, 40].

Finally, a 3 days boiling acid treatment was performed to remove residual surface *sp*^2^ carbon and metal impurities [40–42]. This treatment is also useful for increasing the dispersibility of DNDs [41] by increasing the abundance of oxygen-containing functional groups (e.g., carboxyl and hydroxyl groups) on their surface [42]. The DND powder was transferred into an acid mixture [HNO_3_ / H_2_SO_4_, / H_2_O = 12 / 76 / 12 (wt%)], and ice-bath sonicated (Bioruptor UCS-200TM, Cosmo Bio) for 10 min to achieve a dispersed state (on / off = 30 s / 30 s). The mixture was stirred and heated at 130 °C for 3 days under reflux inside a fume-hood. DNDs were then diluted and cleaned with DI water three times at 150,000 *g* for 20 min to remove metal impurities dissolved in the acid with the aid of an ultracentrifuge (OPTIMA TLX and TLA-110 rotor, Beckman Coulter) and an ultrasonic disruptor (UR-21P, Tomy).

The PL signals from the Sn-doped DNDs were measured using a custom-built confocal microscope equipped with a Czerny–Turner monochromator (Acton SP2300i and SPEC-10, Princeton Instruments) [30]. Approximately 15 µL of sample solution with a concentration of ~ 1 mg/mL was deposited onto a glass coverslip and allowed to dry in air. An excitation laser beam with a wavelength (*λ*) of 532 nm was reflected by a 552 nm long-pass dichroic mirror and focused onto the sample from the backside of the coverslip using an oil-immersion objective lens with a numerical aperture of 1.45. The typical laser power was set to 200 µW, as measured before the objective lens. The PL signal from the sample was collected by the same objective and then passed to the dichroic mirror and a 40 µm pinhole. The PL signal was then directed to one of two paths. The first path was used for PL image measurements or photon autocorrelation measurements based on a Hanbury, Brown, and Twiss method [43–45]. The PL signal was passed through a 620 ± 5 nm band-pass filter (HMX0620, Asahi Spectra) and then split by a half-beam splitter and directed to two single-photon counting modules (SPCM-AQRH-14, Excelitas Technologies). The pulse signals from the two modules were sent to a time-to-digital converter (Time Tagger 20, Swabian Instruments). To acquire PL images, the objective lens, mounted on a three-axis piezo stage, performed raster scanning at the appropriate axial position. During the scanning process, the PL intensity was monitored by summing the signals from the two modules. To acquire the autocorrelation function, the correlation rate between the two signals was measured as a function of the time delay *τ* between the pulse arrival times from the two modules. In the second path, a mirror on a 90° flip mount placed before the band-pass filter directed all signals to the monochromator for PL spectrum acquisition. A grating with a groove density of 150 gr/mm was selected in the monochromator for observation of the entire spectral shape. The spectral wavelength range was ~ 500 nm, with a spectral resolution of ~ 0.6 nm. All the measurements were performed under ambient conditions.

## Results and discussion

Figure 1d shows a PL image of the Sn-doped DNDs. With the aid of the band-pass filter, a well-dispersed bright spot (Spot A) was observed. Although the spot shape became oval, likely because of the imperfect alignment of the optical system, the spot size was well-focused and close to the diffraction-limited spot size. The upper plot in Fig. 1e shows the PL spectrum acquired at Spot A. A sharp peak with a wavelength of ~ 620 nm was observed in the broad spectral band ranging from 550 to 950 nm. Several bright spots with sharp peaks at ~ 620 nm, similar to Spot A, were also identified and labeled as Spots B–E. The PL spectra of these spots are also shown in Fig. 1e. The average peak wavelength for Spots A–E was 620.3 nm with a standard deviation of 1.9 nm, and the average spectral width was 5.7 nm with a standard deviation of 1.7 nm. The peak wavelengths and spectral widths of Spots A–E are consistent with those reported in previous studies [19, 33–36, 46–49]. Hence, the sharp peaks are attributed to the ZPL of SnV^−^. However, the broad spectral band ranging from 550 to 950 nm corresponds to the PL signals from the DNDs themselves and NV centers, which are generated by the nitrogen atoms present in the DND explosives [40]. Although these bright spots occasionally exhibited photoblinking—a behavior that has also been reported for single SnV centers in bulk diamond [35, 36]—the typical photon counts for Spot A–E ranged from 50 to 100 kcount/s under a laser power of 200 µW.

**Fig. 2:**
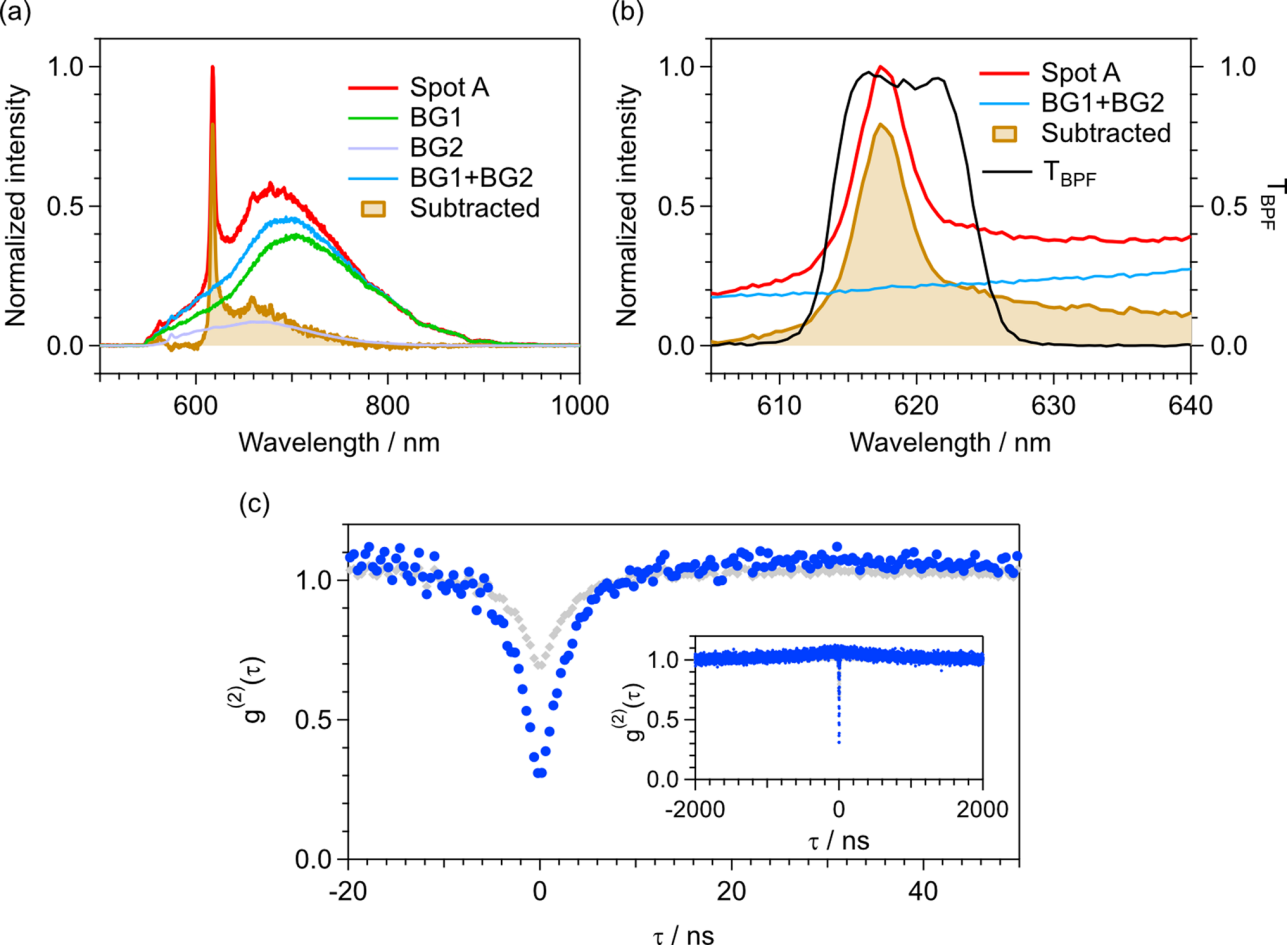
**a** Background (BG1 + BG2)-subtracted spectrum corresponding to Spot A. BG1: PL spectrum of DND without the SnV center’s ZPL. BG2: PL spectrum of NV^0^. **b** Enlarged view of figure (**a**) in the horizontal direction. The transmittance curve for the 620 ± 5 nm band-pass filter (*T*_BPF_) is also shown. **c** Photon autocorrelation measurements *g*^(2)^(*τ*) as a function of time delay *τ* for Spot A, showing data before (gray diamonds) and after (blue circles) the background correction. The inset shows *g*^(2)^(*τ*) in the range from − 2000 to 2000 ns

To clarify the spectral shape of the SnV^−^ in DNDs, we subtracted a background signal from the PL spectrum of Spot A (Fig. 2a, b). A PL spectrum without the ZPL of SnV^−^ was obtained at another bright spot in a PL image and used as background BG1 in the initial analysis. Because the typical spectrum of the SnV^−^ exhibits no PL signal beyond 800 nm [19, 33–35, 46–48], the BG1 was scaled by a constant factor and subtracted from the PL spectrum of Spot A to minimize the average intensity in the range 800–950 nm. Nonetheless, the subtracted spectrum still shows a shoulder in the 550–600 nm range. Within this wavelength region, the signal is known to be dominated by the neutral NV center (NV^0^) rather than the NV^−^ center [34, 50], suggesting that the initial subtraction is insufficient. Hence, a PL spectrum of NV^0^ obtained from an NV center ensemble in a high-pressure, high-temperature bulk diamond was used as background BG2 to further correct the spectrum. Figure 2a, b show the background-subtracted spectrum obtained using the sum of BG1 and BG2 to minimize the intensities in the 550–600 nm and 800–950 nm ranges. The overall spectral shape, including the ZPL and the phonon sideband (PSB), is highly consistent with those of previously reported SnV centers in bulk diamonds [19, 33–36, 46–49].

We measured the photon autocorrelation function *g*^(2)^ to investigate the presence of a single SnV^−^ in a DND. Figure 2c shows the *g*^(2)^ function for Spot A, which exhibits a rapid increase followed by a gradual decay to a constant value at a long time delay |*τ*| because of antibunching and bunching effects. The *g*^(2)^ function was normalized to *g*^(2)^(*τ* → ∞) = 1 by fitting using the function

1 $$\begin{array}{c}{g}^{\left(2\right)}\left(\tau \right)=1-{\alpha }_{1}exp\left(-\frac{\left|\tau \right|}{{\tau }_{1}}\right)+{\alpha }_{2}exp\left(-\frac{\left|\tau \right|}{{\tau }_{2}}\right),\end{array}$$

where *τ*_1_ and *τ*_2_ are the antibunching and bunching time constant, respectively, and *α*_1_ and *α*_2_ are the degrees of antibunching and bunching, respectively (ideally *α*_1_ = 1 + *α*_2_). If *g*^(2)^(0) < 0.5 after normalization, then the target is a single-photon emitter [33, 43–45]. In our case, a clear decrease in *g*^(2)^ was observed at *τ* = 0 ns. However, the measured *g*^(2)^(0) was 0.68, exceeding the threshold of 0.5. This result is attributed to the background signal overlapping with the signal of SnV^−^ (Fig. 2a). In this case, *g*^(2)^(0) can be evaluated after background correction using the background-corrected *g*^(2)^ function given by the relation

2 $$\begin{array}{c}{g}^{\left(2\right)}\left(\tau \right)=\frac{{g}_{\text{raw}}^{\left(2\right)}\left(\tau \right)-\left(1-{\rho }^{2}\right)}{{\rho }^{2}},\end{array}$$

where ${g}_{\text{raw}}^{\left(2\right)}\left(\tau \right)$ is the raw data and *ρ* = *S*/(*S* + *B*), where *S* and *B* are the signals of the SnV^−^ and the background, respectively [33, 43–45]. In this experiment, *S* and* B* were evaluated using the subtracted spectrum, the background spectrum, and the transmission curve for the band-pass filter (*T*_BPF_) (Fig. 2b). For each spectrum, the* T*_BPF_ was applied and the spectral areas were integrated over the range from 605 to 635 nm. By comparing the areas of *S* and *B*, we determined the value of *ρ* to be 0.66. The background-corrected *g*^(2)^ function obtained using relation (1) is shown as blue circles in Fig. 2c. Finally, by applying fitting function (2), we calculated *g*^(2)^(0) to be 0.27, with a fitting error of 0.01. This result provides clear evidence that a single-photon source originating from a single SnV^−^ was found in a DND.

**Fig. 3:**
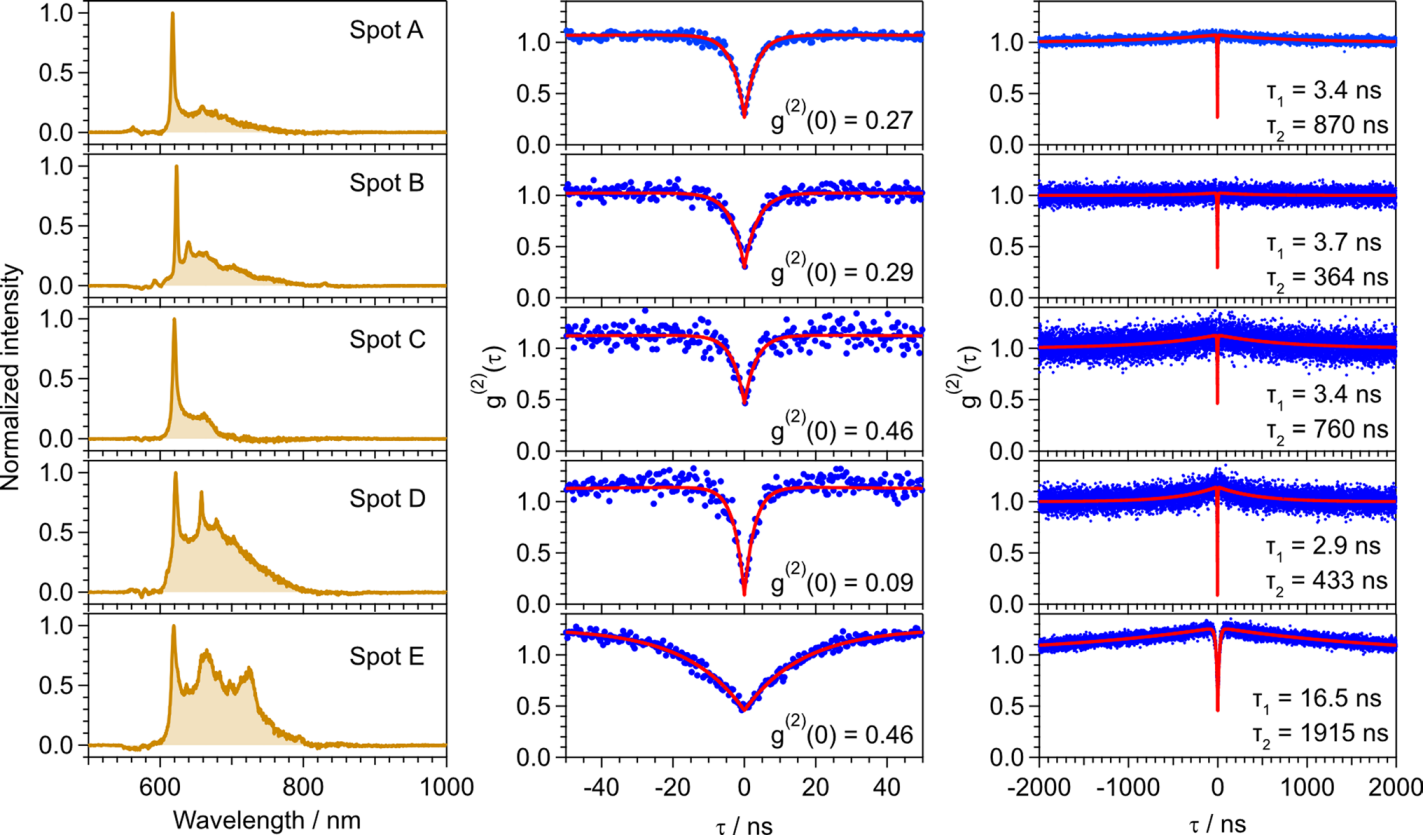
(left) Background-corrected spectrum of each bright spot. The intensity was re-normalized after the correction. (center) The *g*^(2)^ function after the background correction displayed in the range − 50 ≤ *τ* ≤ 50 ns. (right) The *g*^(2)^(*τ*) function from − 2000 to 2000 ns. The time bin is 0.4 ns. The typical measurement time is 10,000 s. The blue dots are experimental data, and the red curve is the fitting result. The values of *g*^(2)^(0), *τ*_1_, and *τ*_2_ after the fitting are shown in the plots of the *g*^(2)^ function

Figure 3 summarizes the background-corrected PL spectra and *g*^(2)^ functions for Spots A–E analyzed using the same procedure described in Fig. 2. The PL spectra corresponding to Spots A–C show good agreement with the reported PL spectra of SnV^−^ centers in bulk diamonds (left column of Fig. 3). For the PL spectra corresponding to Spots D and E, although the spectral component at longer wavelengths (650–800 nm) remains, the ZPL peak wavelength and width are also consistent with the results of previous studies (Fig. 1e). The *g*^(2)^(0) values for all the bright spots were found to be less than 0.5 (center and right columns of Fig. 3). The *g*^(2)^(0) values for Spots A, B, and D are sufficiently low to indicate the existence of single SnV^−^ centers. The *g*^(2)^(0) values for Spots C and E remain close to 0.5 even after background correction, likely because of photoblinking and residual background signals, although the possibility of multiple SnV^−^ centers cannot be ruled out. The *τ*_1_ and *τ*_2_ values show noticeable differences between Spots A–D and Spot E. The average values of *τ*_1_ and *τ*_2_ for Spots A–D are 3.4 and 607 ns, with standard deviations of 0.3 and 213 ns, respectively. The *τ*_1_ and *τ*_2_ are associated with the lifetime of the excitation states and shelving states. Although *τ*_1_ and *τ*_2_ depend on the laser power [36], the *τ*_1_ values for Spots A–D are slightly shorter than those typically reported for bulk diamonds (4 ≤ *τ*_1_ ≤ 6 ns) [33, 35, 36, 48]; by contrast, the *τ*_2_ values are substantially longer than the reported values (40 ≤ *τ*_2_ ≤ 220 ns) [35, 36, 48]. These differences might be related to the distance between the SnV^−^ position and the particle surface, the surface condition, and the difference in surrounding environments between the DND particles and the bulk diamond. The *τ*_1_ and *τ*_2_ values for Spot E are substantially greater than those for Spots A–D. However, such a long *τ*_1_ has also been reported for single SnV^−^ centers created near the surface of a bulk diamond after an annealing procedure [47]. The authors of this previous study reported that *τ*_1_ for these single SnV^−^ centers ranges from 7 to 25 ns, with the variation of *τ*_1_ likely related to the damage on the diamond surface induced by the annealing process. Hence, in our case, the surface damage and surrounding environments of the DND for Spot E may differ from those for Spots A–D.

The SnV^−^ centers were hosted in Sn-doped DNDs with a mean particle size of ~ 5 nm. It is important to note that the size distribution of DNDs containing SnV^−^ centers remains unclear. Since there are DND particles with a diameter of > 10 nm as shown in Fig. 1c, it is possible that the SnV^−^ centers may only exist in these larger particles. In addition, the estimation of the concentration of SnV^−^ center in DNDs is a challenging task. In the case of NV^−^ centers in DNDs, the concentration of NV^−^ can be estimated via their forbidden transition in electron paramagnetic resonance (EPR) measurements thanks to their *S* = 1 electron spin property [40, 41]. In the case of negatively-charged group IV color centers (*S* = 1/2), their EPR signals overlap with the signals from other defects, such as dangling bonds and paramagnetic substitutional nitrogen defects (P1 centers) [51, 52]. As a result, currently there are no established methods for estimating SnV^−^ concentration in DNDs. Although the concentration may also be estimated from their fluorescence ratio between SnV^−^ and NV^−^ in PL spectroscopy, this is also difficult due to their close proximity to the surface which can trigger photoblinking and photobleaching effects.

Given these challenges, the SnV^−^ concentration in this work was roughly estimated as follows. We used the NV^−^ concentration reliably estimated through EPR for Sn-doped DNDs, which stands at 0.35 ppm—consistent with our previous investigation on Si-doped DNDs [40]. To our knowledge, this NV^−^ concentration is the highest among reported DNDs without electron-irradiation and annealing processes [40, 41, 52], and corresponds to one NV^−^ center per 250 particles of 5 nm DND (in Ref. [40], we estimated that the concentration corresponds to one NV^−^ center per 22 particles of  11.2 nm Si-doped DND). Confocal imaging was performed on more than 600 spots, of which approximately 30 spots (~ 5% of the measured spots) showed ZPL of SnV^−^, while the remaining spots likely contained only NV centers. By combining the mean particle size data (5 nm), the NV^−^ concentration, and the SnV/NV spot ratio, we estimated the SnV^−^ concentration to be about 0.018 ppm (i.e., one SnV^−^ center per 6000 particles of 5 nm DND). However, this estimation is based on the assumption that each SnV-containing confocal spot exhibits a one-to-one ratio of NV^−^ to SnV^−^ centers. In consideration of the SnV^−^ fluorescence intensity versus NV^−^ fluorescence intensity from PL spectra like Fig. 1e, the number of NV centers should be greater than that of SnV centers. If there are 10 NV^−^ centers versus one SnV^−^ center on average in such a spot, the estimation makes up to one SnV^−^ center per 60,000 particles of 5 nm DND. It is also important to note that the quantum yields of NV^−^ and SnV^−^ are approximately the same [17], but the difference in absorption coefficients for 532 nm laser excitation has not been taken into account.

Although generating single SnV centers in single DNDs is crucial for providing single-photon sources for quantum applications, increasing the yield of SnV centers is equally important for both quantum and biological applications. To increase the number of SnV centers, it is essential to optimize both the detonation and post-detonation processes. Regarding the post-detonation process, it is important to note the peaks observed other than the ZPL of SnV^−^, specifically those at 593 nm [33, 36, 46, 48, 49], 631 nm [35–37, 48], 647 nm [19, 33–37, 48, 49], and 663 nm [37, 38, 48]. These peaks are believed to be associated with Sn-related defects, although their exact nature remains uncertain. Interestingly, some of these peaks can be converted to the ZPL of SnV^−^ after appropriate post processing. For example, Corte et al. reported the peak wavelength distribution under two different surface conditions for Sn-implanted bulk diamonds [35]. One condition was thermal annealing after Sn-ion implantation, and the other was oxygen termination by an additional oxygen plasma treatment. The peak wavelength is mainly distributed at 647 nm in the former case, whereas the distribution shifts to 620 nm is the latter case. This result may indicate stabilization of the charge state of the SnV center as a result of the change in surface conditions. Note that, as previously described by Corte et al.[35], the interpretation that the peak at 647 nm corresponds to the neutral charge state of the SnV center (SnV^0^) appears to be ruled out by the results of a recent study [48]. In another example, Cheng et al. [46] performed femtosecond laser annealing of Sn-ion-implanted diamond to create site-selective single SnV centers. After appropriate laser irradiation of Sn-ion-implanted diamond, the sharp peak at 595 nm disappeared and the ZPL of the SnV^−^ emerged at 620 nm. On the basis of laser irradiation experiments and density functional theory calculations, they hypothesized that the peak at 595 nm corresponds to an SnV defect bound to a carbon self-interstitial, which serves as a precursor state to a stable SnV^−^ center. Subsequent laser treatment would lead to migration of the carbon interstitial, thereby switching to a stable SnV^−^ center with a ZPL at 620 nm.

**Fig. 4:**
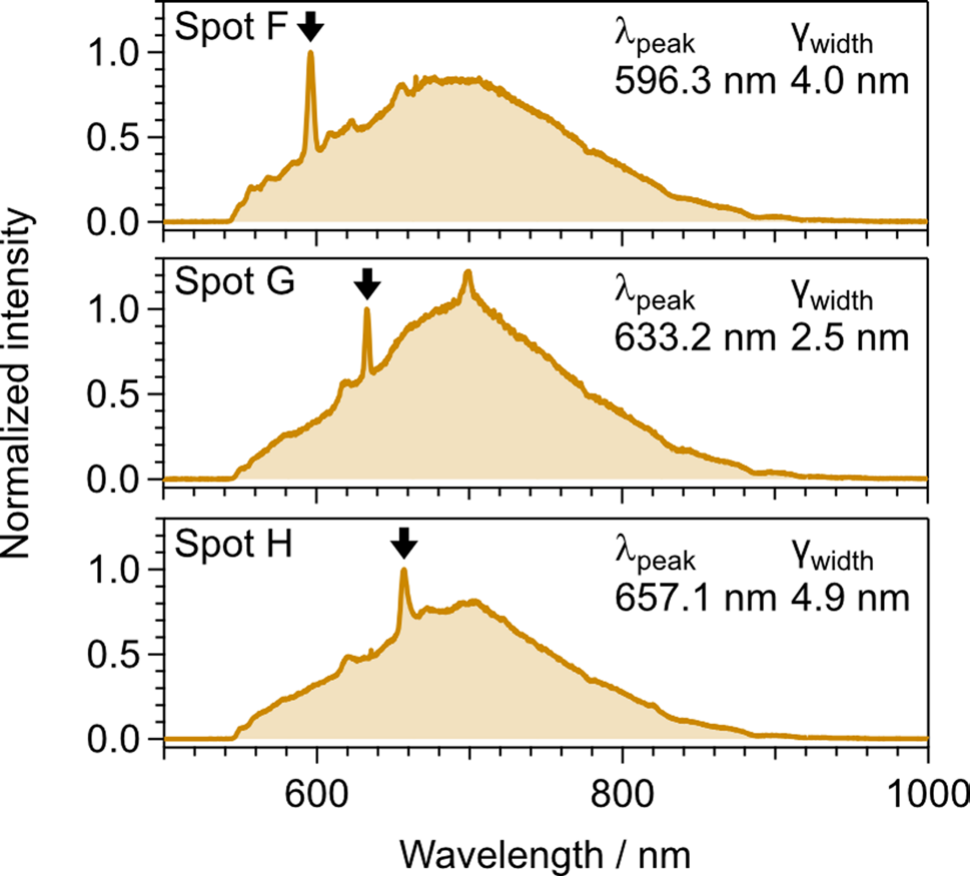
PL spectra with sharp peaks other than the ZPL of SnV centers. The *λ*_peak_ and γ_width_ were obtained using the same method as described in Fig. 1e, but the fitting wavelength ranges were changed to 585–605 nm (Spot F), 620–640 nm (Spot G), and 645–665 nm (Spot H)

In our case, the DND surface after the boiling acid treatment is terminated by oxygen-containing derivatives, such as carboxyl groups [42], which can facilitate charge stabilization of color centers or surface functionalization. An additional surface treatment such as oxygen plasma treatment may further stabilize SnV^−^ centers. In addition, several bright spots with sharp peaks (except for the peak at 620 nm) were observed (Fig. 4). Notably, the spectrum corresponding to Spot F shows a sharp peak at 596 nm, which may correspond to the aforementioned SnV-carbon interstitial. The peaks corresponding to Spots G (633 nm) and H (657 nm) may also be related to the reported peaks at 631 nm and 663 nm, suggesting that further post-detonation processing would enhance the ZPL of SnV^−^.

In the previous and current studies, the generations of SiV, GeV, and SnV centers in DNDs were confirmed. Finally, the possibility of generating PbV center, the last group IV color center, in DND particles should be mentioned. The PbV center exhibits the shortest ZPL wavelength (~ 550 nm) and the longest electron spin coherence time among the group IV color centers [17]. However, generating PbV centers is highly challenging due to the largest atomic size of Pb among group IV elements. The high toxicity of Pb atoms will also pose a significant problem for biological applications. However, several studies have reported the successful generation of PbV centers for quantum applications, such as through Pb ion implantation and subsequent high-temperature annealing under high pressure in bulk diamond [53]. In addition, the generation of PbV centers was also performed during the diamond synthesis from a Pb-graphite system using a HPHT technique [54]. Considering these studies, PbV centers could potentially be generated using a detonation technique.

## Conclusion

The successful generation of SnV centers in Sn-doped DNDs was demonstrated with the aid of the 3 days boiling acid treatment and optical measurements using a customized confocal system. The detonation technique has opened a new pathway for synthesizing SnV-containing NDs. The mean particle size of the Sn-doped DNDs was ~ 5 nm. Although the DNDs containing SnV^−^ centers may be larger than the mean particle size, we consider these SnV-containing DNDs to be the smallest among SnV-containing NDs. Their small size makes them highly suitable for imaging and sensing at local positions within cell organelles. In addition, single SnV centers were measured in DNDs, which can potentially be used as single-photon sources in quantum devices. Higher SnV generation efficiency is essential for both efficient single-photon emitter production and brighter DND sensors in biological applications. Investigations into improvements in the detonation and post-detonation processes are expected to further enhance the capabilities of DND sensors across multiple fields.

## Data Availability

The data that support the findings of this study are available from the corresponding author upon reasonable request.
